# Chronic suppurative otitis media causes macrophage-associated sensorineural hearing loss

**DOI:** 10.1186/s12974-022-02585-w

**Published:** 2022-09-12

**Authors:** Anping Xia, Anthony Thai, Zhixin Cao, Xiaohua Chen, Jing Chen, Brian Bacacao, Laurent A. Bekale, Viktoria Schiel, Paul L. Bollyky, Peter L. Santa Maria

**Affiliations:** 1 Department of Otolaryngology-Head and Neck Surgery, School of Medicine, Stanford University, Palo Alto, CA 94305 USA; 2 Department of Pathology, Shandong Provincial Hospital Affiliated to Shandong First Medical University, Jinan, Shandong Province China; 3 Department of Otolaryngology-Head and Neck Surgery, the First Affiliated Hospital, Zhengzhou University, Zhengzhou, Henan Province China; 4 Department of Medicine, Infectious Diseases, Stanford University, Stanford, Palo Alto, CA 94305 USA

**Keywords:** CSOM, PA, SNHL, Macrophages, HC loss, Cytokines

## Abstract

**Background:**

Chronic suppurative otitis media (CSOM) is the most common cause of permanent hearing loss in children in the developing world. A large component of the permanent hearing loss is sensory in nature and our understanding of the mechanism of this has so far been limited to post-mortem human specimens or acute infection models that are not representative of human CSOM. In this report, we assess cochlear injury in a validated *Pseudomonas aeruginosa* (PA) CSOM mouse model.

**Methods:**

We generated persisters (PCs) and inoculated them into the mouse middle ear cavity. We tracked infection with IVIS and detected PA using RT-PCR. We assessed cochlear damage and innate immunity by Immunohistochemistry. Finally, we evaluated cytokines with multiplex assay and quantitative real-time PCR.

**Results:**

We observed outer hair cell (OHC) loss predominantly in the basal turn of the cochlear at 14 days after bacterial inoculation. Macrophages, not neutrophils are the major immune cells in the cochlea in CSOM displaying increased numbers and a distribution correlated with the observed cochlear injury. The progression of the morphological changes suggests a transition from monocytes into tissue macrophages following infection. We also show that PA do not enter the cochlea and live bacteria are required for cochlear injury. We characterized cytokine activity in the CSOM cochlea.

**Conclusions:**

Taken together, this data shows a critical role for macrophages in CSOM-mediated sensorineural hearing loss (SNHL).

**Supplementary Information:**

The online version contains supplementary material available at 10.1186/s12974-022-02585-w.

## Introduction

CSOM is a neglected tropical disease that affects around 330 million people worldwide and is the most common cause of permanent hearing loss among children in the developing world [1]. It is characterized by a chronically discharging and infected middle ear. There is currently no effective cure.

Among CSOM pathogens, PA is the leading culprit [2]. PA colonizes the middle ear via a perforation in the tympanic membrane and establishes itself into a biofilm community, complicating attempts to treat and fully eradicate the infection [2, 3]. Over the course of the disease, the infection waxes and wanes as the population of bacteria within the biofilm responds, in part, to immune attack or topical antibiotic therapy.

PA infection in CSOM is caused by PCs [4]. These are metabolically inactive bacteria that are less susceptible (more tolerant) to many antibiotics [5]. PCs are phenotypically distinct from “free swimming” planktonic cells (exponentially growing cells) that are typically cultured by ear swabs [6].

While CSOM is known to cause conductive hearing loss, which is potentially reversible, several groups have provided evidence that the inner ear is also affected causing the more permanent and impactful SNHL [7, 8]. The human post-mortem findings are cochlea hair cell (HC) loss and a reduced size of the stria vascularis, particularly in the basal turn. This affected region matches the clinical findings of high-frequency SNHL in CSOM patients [9–11]. Our poor understanding of the mechanisms underlying PA-mediated SNHL in CSOM hinders development of therapies to prevent hearing loss in hundreds of millions globally.

To date, most of our knowledge of how the inner ear responds to infectious disease is in animal models of acute otitis media. In these models, there is gross bacterial invasion into the inner ear and a massive exposure of ototoxins including endo- or exotoxins, virulence factors, peptidoglycan fragments, teichoic acids and hydrolytic enzymes [12]. These rapid, widespread, grossly disruptive changes do not match the cochlear injury patterns in human CSOM patients, nor the clinical presentation of high-frequency SNHL.

To overcome this gap, we recently developed and validated a CSOM animal model that represents the human disease [13]. Using this model, we began a stepwise investigation to understand how SNHL develops in CSOM.

## Materials and methods

### Animals and ethics approval

All animal procedures were approved by the Institutional Animal Ethics Committee (IACUC) at Stanford University. 6- to 8-week-old wild-type CBA/CaJ mice (Ca# 000654 The Jackson Laboratories, Bar Harbor, ME USA) were used for all experiments and housed in the Stanford University animal care facility with ad libitum access to food and water. Mice procedures were performed under anesthesia using ketamine (80–100 mg/kg) and xylazine (8–10 mg/kg).

### Preparation of PA and PCs

Frozen glycerol stocks of PAO1 (A gift from Dr. Robert E. Hancock Lab in University of British Columbia, Vancouver, BC, Canada) were plated on Luria–Bertani (LB) agar and grown overnight at 37 °C. All organisms were then cultured in LB from individual colonies at 37 °C, shaking at 200 rpm. Cultures were placed again on LB plates. An isolated colony from the second agar plate was picked and grown overnight at 37 °C in 10 mL of LB under shaking, aerobic conditions. *P. aeruginosa* PAO1 with constitutive expression of a chromosomal-encoded luminescence reporter (PAO1.lux) was constructed as previously described [14]. The MIC of the ofloxacin was determined against PAO1 using the broth microdilution method as previously described [4]. The bacteria were grown overnight at 37 °C in LB medium against serial dilution method (twofold), in a 96-well polypropylene microplate. Next, the bacteria growth in presence of the drugs was evaluated by visual observation of the solution (clear or cloudy) in the wells. The MIC (0.96 μg/mL) was obtained from the lowest concentration of the drug which show no bacterial growth. To generate PCs, bacteria were grown for 30 h to reach the stationary phase. Bacteria were then treated with 5 μg/mL of ofloxacin (FLOXIN®Otic) for 5 h and washed three times in PBS via centrifugation at 8000×*g* for 5 min. The stock was resuspended in PBS. Bacterial concentration (CFU/mL) was measured by plating 100 μL of serial stock dilutions on LB agar plates, colonies were counted at 48 h after plating. Prior to each in vivo experiment, the CFU/mL was measured to ensure the concentration had not changed. A concentration of 1.63 × 10^7^ CFU/mL was used for in vivo inoculations. For experiments involving inoculation of PA supernatant, stationary phase PA were generated as above and left untreated. The bacterial solution was passed through a 0.2-µm filter to remove PA. A cultured plate was incubated at 37 °C for > 48 h to ensure no bacteria were present. For inactivation of PA, the tube containing the stationary phase bacteria was placed in water bath at 100 °C for 40 min to kill live bacteria which significantly reduces lipopolysaccharide (LPS) activity as previously described [15]. To ensure that all bacterial cells were killed, a sample was taken after the above process and a cultured plate was incubated for > 48 h with no resulting bacterial growth.

### PC identification

For the survival assay, stationary phase PA cultures were treated with ofloxacin at 5 µg/mL. At indicated timepoints (0, 1, 2, 3, 4, 5 and 24 h), 1-mL aliquots were removed and washed 3 times with PBS. Samples at each time point were then serially diluted and inoculated onto LB agar plates for 48 h in triplicates to determine the concentration in CFU/ml. For growth curves, wild-type PA and PCs were diluted at 1:100 into 200 µL of LB in 96-well microtiter plates. Plates were incubated at 37 °C with constant shaking. OD600 was measured at 15-min intervals using a microplate reader (spectraMax M2, Molecular Devices, Downingtown, PA). For assessment of ATP production, the ATP levels of wild-type PA and PCs were measured in triplicate using a BacTiter Glo kit (# G8230, Promega, Wisconsin, USA.) according to manufacturer instructions. Bicinchoninic acid (BCA) assay was also performed to measure the total protein according to manufacturer instructions (# 23225, Thermo Fisher Scientific, MA, USA). Finally, the ATP level was normalized to the total protein for each sample. For determination of the minimum inhibitory concentration (MIC), the MIC values of ofloxacin were determined for PCs using the broth microdilution method. Bacteria were inoculated at 1:100 into 200 µL of LB containing serially diluted concentrations of the drug in 96-well microtiter plates. Plates were incubated at 37 °C with constant shaking, and OD600 was measured at 15-min intervals using a microplate reader as above. The lowest ofloxacin concentration showing no growth after 24 h was selected as the MIC.

### Chronic suppurative otitis media model

We adapted our validated model of CSOM (Khomtchouk et al. 2020). In brief, mice were anesthetized and placed on the surgical stage under the microscope. Following subtotal TM perforation of the left ear (Additional file 1: Fig. S1), 5 µL of PCs was inoculated into the middle ear cavity (1.63 × 10^7^ CFU/mL). The inoculations were performed from 9 to 10 am in all our experiments. The observation of the middle ear in all the mice at 14 days after inoculation features grade III–IV CSOM as previously described [13]. This same method was employed to inoculate heat-inactivated PA or PA supernatant groups. 5 µL of 1X PBS was inoculated into the middle ear cavity serving as control group. Mice were maintained in a prone position for 30 min following inoculation. The mice were used for all experiments at different time points (Additional file 5: Fig. S5).

### Real-time infection tracking

Disease progression was tracked by capturing images with open emission using a LagoX in vivo imaging system (IVIS, Spectral Instruments Imaging, AZ, USA) as previously described [13]. Briefly, using isoflurane, mice were placed on the right lateral position to expose the left ear (CSOM infected ear) at progressive days after inoculation. Images were initially acquired at 60-s exposure with medium binning. If no signal was detected, mice or inner ears were reanalyzed at 300-s exposure with high binning. Background luminescent signal was subtracted from signal from the area around the ear. Chronic infection was designated as the presence of infection at 7 days after inoculation. Following live mouse IVIS, cochleae were dissected out for IVIS using the same method.

### Histological preparations

Middle ears and cochleae were dissected at 3 days (3 d), 7 days (7 d) and 14 days (14 d) after infection (Additional file 1: Fig. S1). In the control group, the middle ears and cochleae were dissected at 14 days. Dissected specimens were fixed in 4% paraformaldehyde (Electron Microscopy Sciences, Hatfield, PA, USA) at 4 °C overnight. Samples were then decalcified in 0.5 M EDTA (VWR, Radnor, PA) for 48 h at 4 °C, and washed three times in PBS (Fisher Scientific). For whole-mount preparations, the organs of Corti were dissected out from the cochleae under a stereo microscope. The cochlear epithelium was divided into three parts: apex (70–100% from the base), middle (30–70% from the base), and base (0–30% from the base). For cryosection preparation, middle ears and cochleae were immersed in a sucrose gradient (10–30%) and embedded in OCT. Samples were collected in 10-µm sections.

### Immunohistochemistry

Whole mount tissues or cryosections were blocked with 5% donkey serum, 0.1% Triton-X 100, 1% BSA, and 0.02% sodium azide (NaN3) in PBS at pH 7.4 for 1 h at room temperature (RT). Samples were then incubated in primary antibodies overnight at 4 °C. The following primary antibodies were employed: rabbit anti-myosin VIIa (1:200; 25-6790; Proteus BioSciences), goat anti-CD45 (1:100; AF114, R&D systems., Minneapolis, MN, USA), rat anti-F4/80 (1:150, ab6640, Abcam Inc., Cambridge, MA, USA), rat anti-Ly-6G/C (1:100: ab2557, Abcam Inc, USA). The specimens were incubated with secondary antibodies diluted in 0.1% Triton-X 100, 0.1% BSA and 0.02% NaN3 in PBS for 1 h at RT. The secondary antibodies were conjugated with Alexa Fluor 488, 546, and 647 (1:500; A11055, A10040, and A31571, Life Technologies, Carlsbad, CA). After washing with PBS, specimens were mounted in ProLong® Gold Antifade Reagent with DAPI (Cell signaling, #8961 Danvers, MA 01923) and placed under a cover slip. Images were captured using a LSM700 confocal microscope (Zeiss, Germany) at 10X magnification.

### Cytokine and chemokine multiplex assay

The inner ears (*n* = 3) were dissected out and the surrounding soft tissues were removed at 7 days. Inner ears were washed with fresh PBS > 20 times to clean the surface of the cochleae. Each wash procedure was performed carefully in sterilized dishes, which were changed at each wash step. Samples were homogenized mechanically in lysis buffer. This buffer included 1% Triton-X 100 (9002–93-, Sigma-Aldrich, St. Louis, MO, USA), 0.5% NP-40 (FNN0021, Thermo Fisher Scientific, Waltham, MA, USA), 25 mM Tris–HCl pH 7.5 (1185-53-1, Millipore Sigma, Burlington, MA, USA), 100 mM NaCl (764714-5, Sigma-Aldrich), Halt protease inhibitor cocktail (78430, Thermo Fisher Scientific) and phenylmethanesulfonyl fluoride (32998-6, Millipore Sigma). Samples were stored at − 80 °C and cytokine analysis was performed at the Human Immune Monitoring Core (Stanford University) as previous described [16]. Briefly, Mouse 48-plex Procarta kits (EPX480-20834901, Thermo Fisher Scientific) were employed and plates were read using FM3D FlexMap instrument with a lower bound of 50 beads per sample per cytokine. Custom Assay Chex control beads were added to all samples. Each sample was tested in triplicate. MFI was averaged over duplicate wells for each cytokine per sample on each plate.

### RT-PCR and quantitative real-time PCR (qPCR)

The inner ears were dissected out and the surrounding soft tissues were removed at 3 days, 7 days and 14 days after inoculation (Additional file 1: Fig. S1). Samples from control groups were dissected out at 7 days after inoculation. The inner ears were washed with fresh PBS > 20 times to clean the surface of the cochleae as described above. The cochleae were separated from vestibule in RLT buffer from the RNeasy mini kit (74004, Qiagen, Germantown, MD, USA). Samples were then mechanically homogenized, and RNA was extracted following manufacturer protocol. Reverse transcription to complementary DNA (cDNA) was performed using the SuperScript VILO cDNA Synthesis Kit (11754050, Thermo Fisher Scientific)) according to manufacturer protocol. To detect PA by PCR, primers targeting the PA O-antigen acetylase gene were selected and amplified a 232-bp amplicon [17]. qPCR was performed using the CFX Maestro software on the CFX Connect Real-Time PCR System (Bio-Rad, Hercules, CA, USA). For qPCR reactions, 1 µl of 20 ng/µl cDNA were added to a 20 µl reaction using SYBR Green Master Mix (1725271, Bio-Rad). Primers were employed for several cytokines (Primer Table). Samples were run in triplicate and mRNA concentration relative to samples from control mice were calculated after normalization to β-actin and GAPDH.

### Statistical analysis

Statistics were performed using GraphPad Prism 9.0 (GraphPad Software Inc., La Jolla, CA, USA). All values in figures are presented as mean ± standard deviation (SD). For three or more groups, we first performed an ANOVA analysis. For a *P* value < 0.05, we then used *t*-tests to compare pairs of subgroups. We compared data between groups using the unpaired, two-tailed Student’s *t* test. *P* < 0.05 was considered statistically significant.

### Primer Table

**Table:** 

Gene	Primer sequence (5′–3′)
IL-1β	F: TGCCACCTTTTGACAGTGATGA R: TGCCTGCCTGAAGCTCTTGT
IL-6	F: ACAAAGCCAGAGTCCTTCAGAGA R: AGGAGAGCATTGGAAATTGGGGT
IL-10	F: GAAGACCCTCAGGATGCGGC R: GGCCTTGTAGACACCTTGGTCTT
TNF-A	F: ACAAGCCTGTAGCCCACGTC R: GGTGAGGAGCACGTAGTCGG
CCL2	F: CTGTTCACAGTTGCCGGCTG R: AGCTTCTTTGGGACACCTGCT
CCL3	F: CAACCAAGTCTTCTCAGCGCC R: TCTTCCGGCTGTAGGAGAAGC
CXCL1	F: GGCTTGCCTTGACCCTGAAG R: CGTTCACCAGACAGGTGCCA
CXCL2	F: GGTACGATCCAGGCTTCCCG R: GGTACGATCCAGGCTTCCCG
β-Actin	F: CCACTGTCGAGTCGCGTCC R: GCCCACGATGGAGGGGAATA
GAPDH	F: TGTGTCCGTCGTGGATCTGA R: CCTGCTTCACCACCTTCTTGAT
PA01	F: CTGGGTCGAAAGGTGGTTGTTATC R: GCGGCTGGTGCGGCTGAGTC

## Results

### Persister cells generation and characterization

**Fig. 1:**
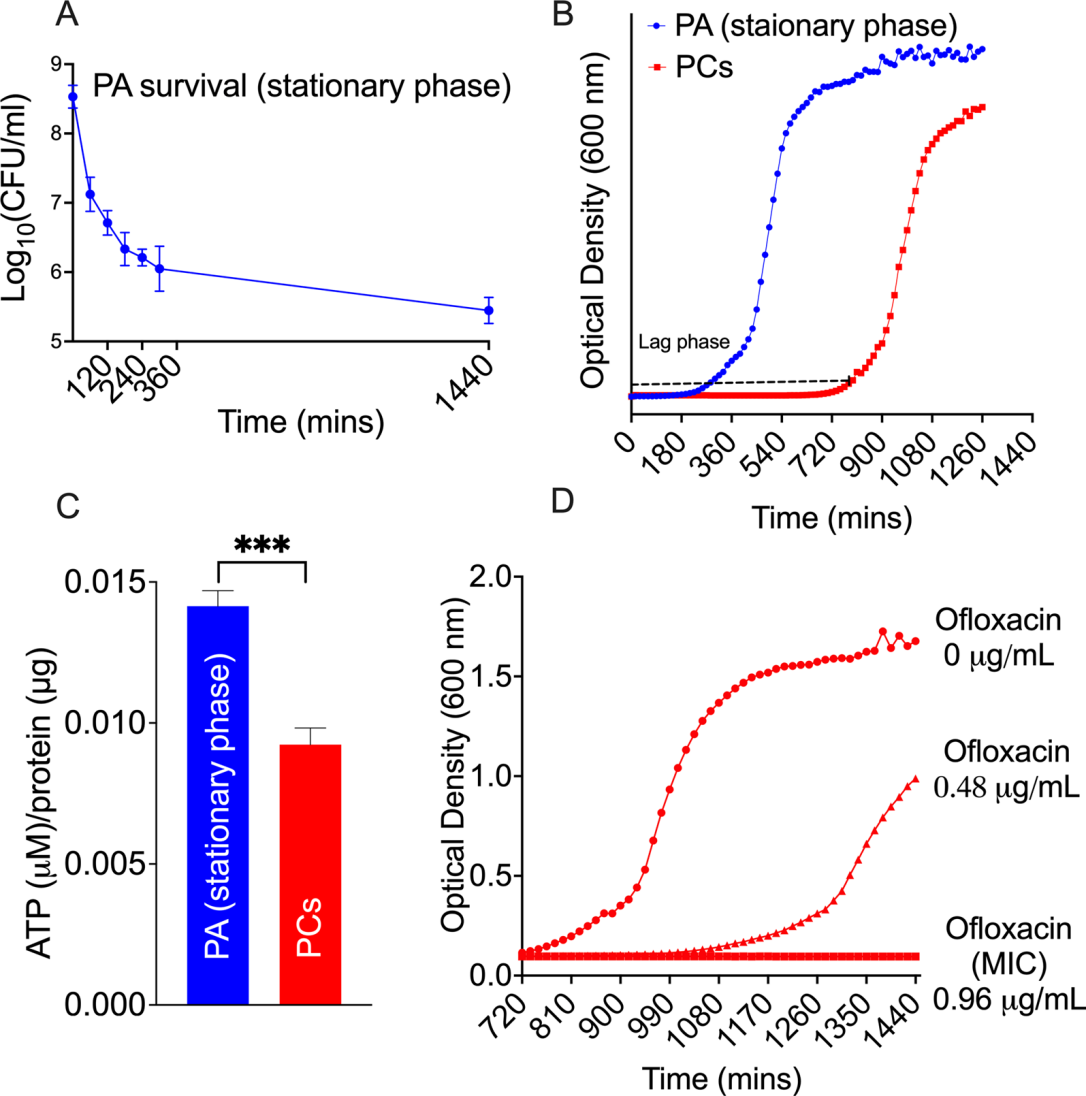
Characterization of persister cells (PCs). **A** Time-dependant survival curve for PA stationary phase after treatment with ofloxacin (5 μg/mL). The survival curve demonstrates rapid killing from 0 to 5 h (predominantly metabolically active cells) and plateauing of survival (PCs) from 5 to 24 h. **B** Growth curve of ofloxacin-generated PCs compared to stationary phase PA. PCs displayed delayed awakening on growth curve compared to stationary phase PA, consistent with the PC phenotype. Upon awakening, the growth rate of the PC phenotype matches that of the stationary phase PA. **C** Intracellular ATP level comparison between PCs compared to stationary phase PA. PCs contained significantly less intracellular ATP following normalization to protein levels compared to stationary phase PA. Due to lower metabolic activity, PC exhibit lower ATP level. **D** The MIC of the surviving PCs was determined by performing growth curves for serial dilutions of ofloxacin (0, 0.48 and 0.96 µg/mL) showing that the PCs are not resistant (MIC < 2 µg/mL)

Human CSOM is often treated with fluoroquinolones, and this likely generates PC phenotypes in CSOM [4]. To mimic the clinical condition, we used a previously described technique to generate ofloxacin induced PA persister cells [18]. Stationary phase PA, cultured in LB media for 30 h, were treated with ofloxacin 5 μg/mL (FLOXIN®Otic) for 5 h. The stationary phase PA were identified by observing the growth curve of cultured PA where the PA concentration declined exponentially from 0 to 5 h after ofloxacin treatment. It neared a plateau by 5 h of treatment, declining slowly from 5 to 24 h (Fig. 1A). Upon reawakening, the lag phase was longer in ofloxacin-treated PA (PCs) than in stationary phase PA, suggesting that ofloxacin-generated PCs awaken at a delayed time point (Fig. 1B). The measured ATP level was significantly lower in ofloxacin-generated PCs than in stationary phase PA (*P* < 0.001) (Fig. 1C). Finally, PCs displayed the same MIC for ofloxacin as stationary phase PA (Fig. 1D). This confirms the PCs are relatively metabolic inactive rather than antibiotic resistant cells.

### OHC loss occurs in the CSOM mouse model

SNHL is mediated by OHC. To better understand the pathophysiology of hearing loss in the current mouse model of CSOM (Additional file 1: Fig. S1), we therefore examined OHC survival following infection. To assess this, we performed immunohistochemistry for myosin VIIa, an ATP-dependent protein expressed on OHC, in whole-mount dissections at 3 days, 7 days and 14 days after PC inoculation using a previously validated technique [19].

**Fig. 2:**
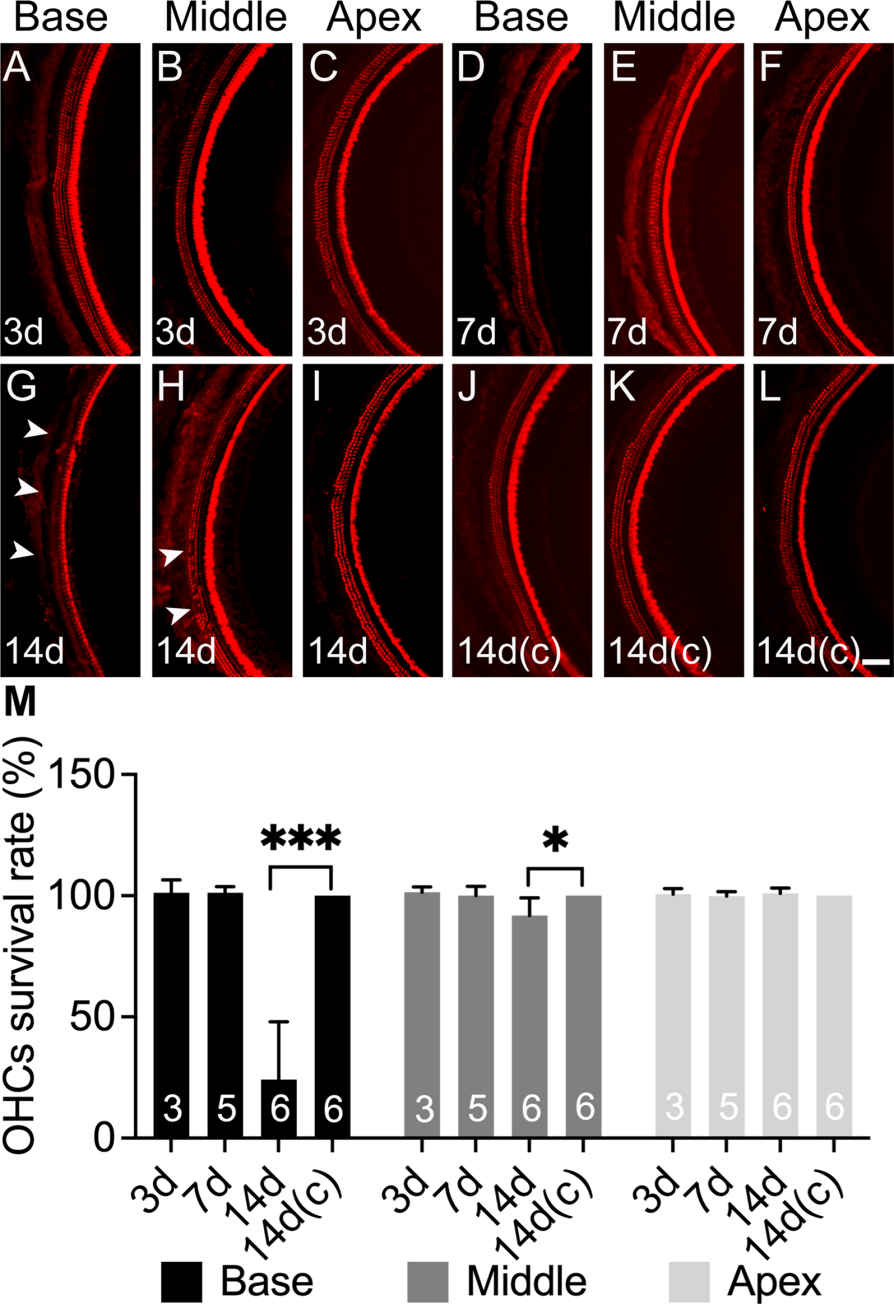
OHC loss occurs in CSOM. **A**–**C** Whole mount immunostaining showed no HC loss.in the base, middle and apex regions of the cochlea at 3 days (**A**–**C**), 7 days (**D**–**F**) and 14 days (**G**–**I**) compared to 14 days control (**J**–**L**). The normal condition displays 3 rows of OHCs on the left and 1 row of IHC on the right of the pictures. CSOM cochlea displayed OHC loss at 14 days (**G**–**I**) in base (**G**, arrowheads) and small areas of absence in middle (**H**, arrowheads), and no OHC in the apex (**I**). **M** OHC survival rate was significantly lower in the base (P < 0.001) and middle (*P* < 0.05) at 14 days compared to control (**M**). The mouse number for each group is presented in the column (M). Red: myosin VIIa. The data represent mean ± SD. Scale bar = 100 µm

We found that no OHC loss occurred at 3 days and 7 days in any specimens (Fig. 2A–F). At 14 days, OHC loss was found predominantly in the basal turn (high-frequency region), with partial OHC loss in the middle turn (middle frequency region) and no OHC loss in the apical turn (low-frequency region) (Fig. 2G–I). To account for differences in cell counts due to dissection variability between samples, we summed the number of missing and surviving OHCs together to obtain the total number of potential OHCs for each cochlea. We then normalized to this total number of OHCs to obtain the OHC survival rate (Fig. 2M). OHC survival rates were 24 ± 23.8% in the base, 92 ± 7.2% in the middle and 100 ± 2.2% in the apex at 14 days. There were significant differences in the base (*P* < 0.001) and middle (*P* < 0.05) turns compared with PBS-inoculated cochleae (control), respectively (Fig. 2J–L, M). The OHC loss pattern in CSOM is similar to those in drug-induced, noise-induced and blast-induced cochlear damage [20–23]. In contrast to OHC loss, no IHC loss was observed in any assessed time points.

We next assessed the requirement for live bacteria in generating SNHL by testing whether heat-inactivated PA could cause hair cell loss (HC). To this end, we inactivated PA using dry heat, a method known to preserve LPS [24]. HC loss was not observed at 21 days following inoculation of heat-inactivated PA (Additional file 2: Fig. S2A). We further tested for the potential for ototoxins released from PA in the middle ear to cause HC loss. Stationary phase supernatant was inoculated into middle ear, and no HC loss was observed by 21 days after inoculation (Additional file 2: Fig. S2B). Together these findings suggest that live replicating bacteria are a requirement of cochlear injury.

### PA does not enter the cochlea in CSOM

**Fig. 3:**
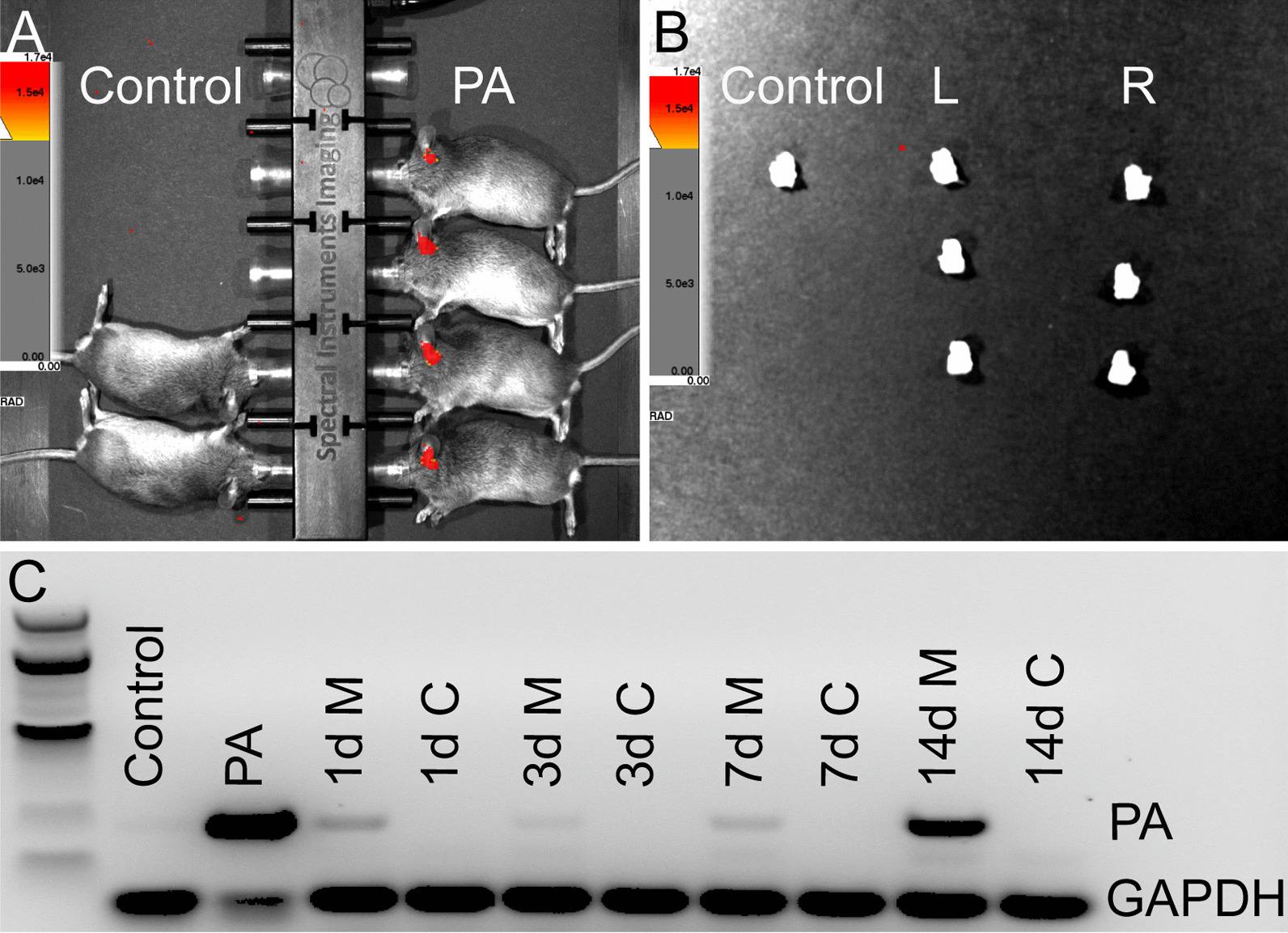
PA is not detected in CSOM cochlea. **A** IVIS revealed the presence of PA (red) in CSOM ears but not in the ears of control mice at 7 days. IVIS was performed with 15 mice for each group. **B** After the cochleae were dissected from the same mice in A, PA was not detected in the inner ears (absence of red) of control or either the infected ear (L) or non-infected ear (R). **C** RT-PCR for the PAO1 O-acetylase gene showed detection of PA in the CSOM middle ears at 1 days (1d M), 3 days (3d M), 7 days (7d M) and 14 days (14d M) but not in the CSOM cochleae at 1 days (1d C), 3 days (3d C), 7 days (7d C) 14 days (14d C) or control cochlea (control). There were 3 mice at each time point. GAPDH was used as housekeeping gene

Our CSOM animal model causes middle ear inflammation with purulent effusion mimicking human CSOM. In this model bacterial counts are highly correlated with luminescence measured by an In Vivo Imaging System (IVIS) [13]. To track PA in the ear, we performed IVIS and RT-PCR. IVIS images revealed that PA was present in CSOM ears at 7 days but not in control ears in vivo (Fig. 3A). We dissected out the inner ear and washed them carefully. No PA was observed in isolated CSOM or control inner ears (Fig. 3B, L and control). The contralateral ears from the CSOM group served as negative control (Fig. 3B, R). Finally, we collected middle ears and cochleae for RT-PCR. PA mRNA was detected in CSOM middle ears from 1 to 14 days. However, no PA mRNA was detected in any cochleae at these time points (Fig. 3C). mRNA extracted from in vitro PA cultures served as a positive control, while mRNA extracted from control cochleae served as a negative control (Fig. 3C). Together, this demonstrates that PA does not enter the cochlea in CSOM.

### Neutrophils are not associated with SNHL

**Fig. 4:**
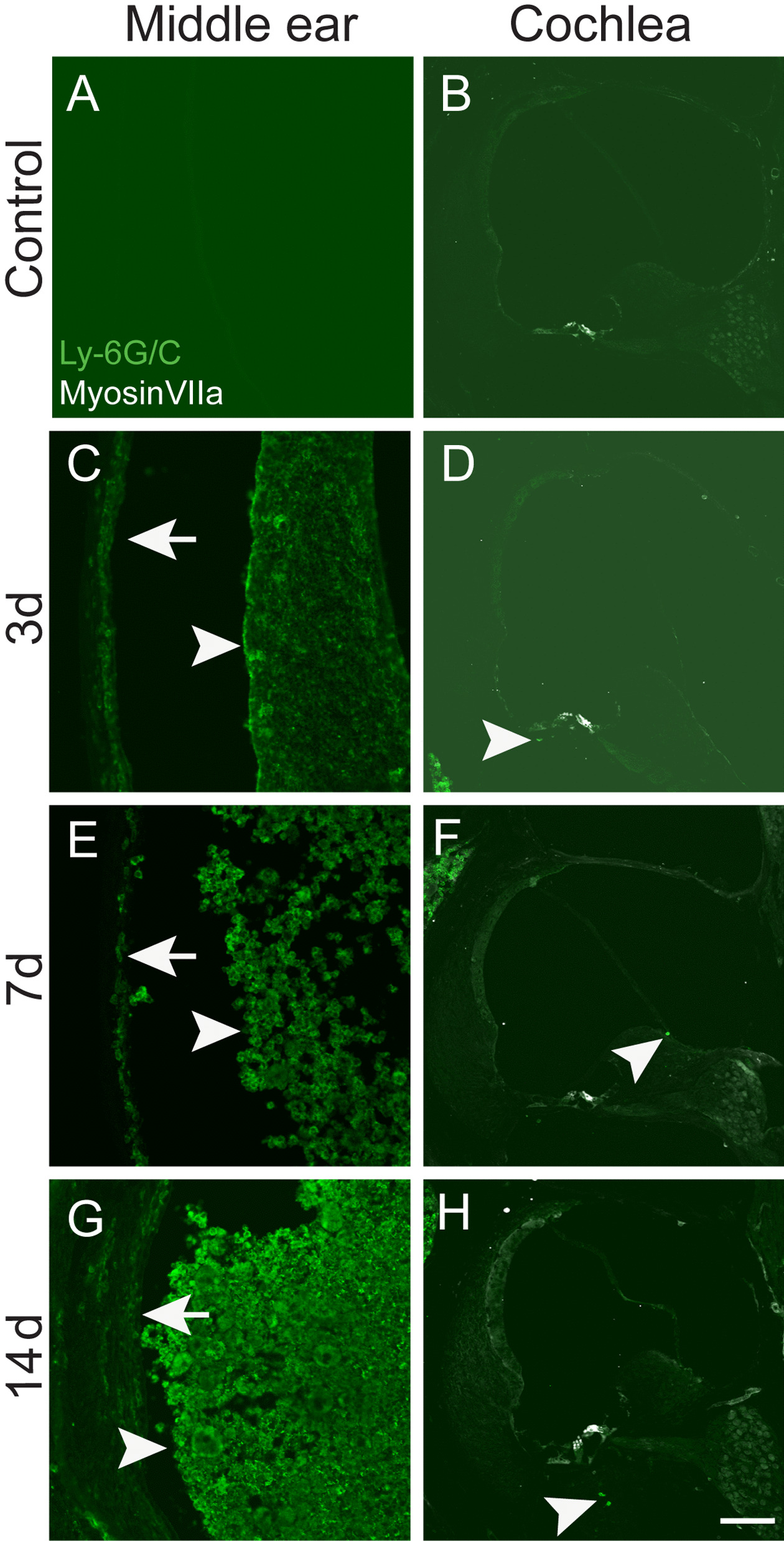
Neutrophils are present in the CSOM middle ear. Staining with the neutrophil-specific marker Ly-6G/C was performed on cryosections of middle ears and cochleae from the same mouse. The CSOM middle ear displayed abundant neutrophils at 3 days, 7 days and 14 days in the effusion (arrowheads to right in **C**, **E** and **G**) and middle ear mucosa (arrows to left in **C**, **E** and **G**), whereas a few limited neutrophils were present in the CSOM cochleae (arrowheads in **D**, **F** and **H**). Top row (**A**, **B**) is control. There were 3 mice at each time point. Scale bar = 100 µm

Neutrophils are responsible for both host defense and host tissue damage in the early phase of bacterial infections particularly in CSOM [25]. We have previously identified that neutrophils were the most abundant cell type in CSOM middle ear effusions using Ly6G/C, a surface marker of mature neutrophils, by multi-parameter flow cytometry (FCM) analysis [25]. In the current study, we performed immunostaining with the Ly6G/C antibody to assess neutrophil presence in the cochlea. Neutrophils were present extensively in the middle ear mucosa (Fig. 4C, E and G arrows) and effusion mass (Fig. 4C, E and G, arrowheads) at 3 days, 7 days and 14 days. Interestingly, only a few neutrophils were present in cochleae at the same time points (Fig. 4D, F and H arrowheads), suggesting that neutrophils are not directly associated with OHC loss in the cochlea.

### Macrophages are the key immune cells found in the CSOM cochlea

**Fig. 5:**
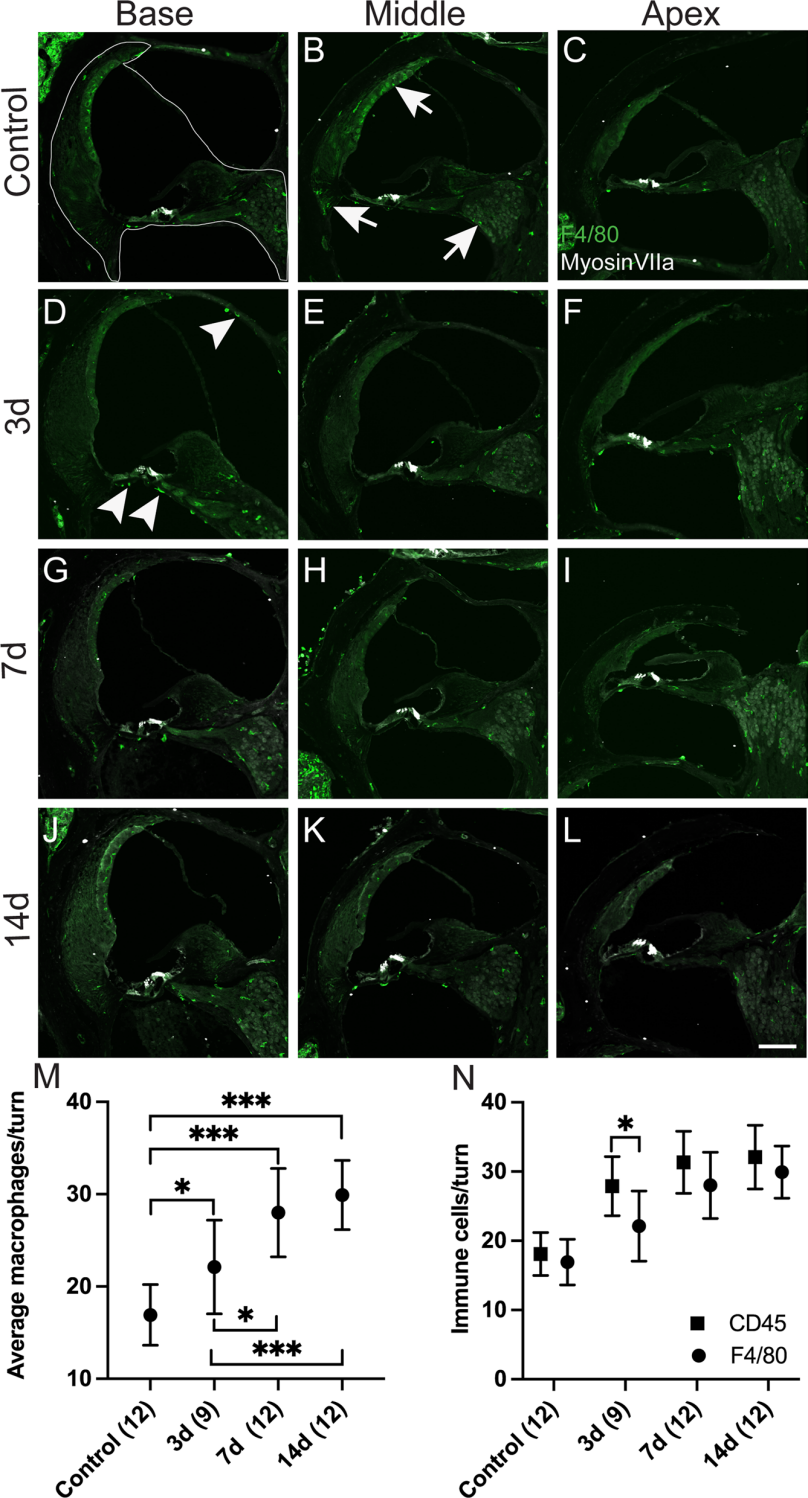
Macrophage numbers are significantly increases in the CSOM cochlea. Cochlear cryosections were stained with the macrophage-specific marker F4/80 (green) and were counted in the region of the spiral ligament, the organ of Corti, spiral ganglion neurons, and the scala media as outlined (A-dotted). Myosin VIIa staining (white) labels HCs in the cochlea. Counts were performed in all cochlear turns (base, middle, apex) in control mice (**A**–**C**) and CSOM cochleae at 3d (**D**–**F**), 7d (**G**–**I**), and 14 DAYS (**J**–**L**). Arrows in the control group (**B**) show macrophages in the stria vascularis, spiral ligament and spiral ganglion neuron area. Arrowheads in CSOM at 3d (**D**) show macrophages also present in the basilar membrane, scala vestibuli. **M** Statistical analysis of the macrophages per turn, revealed significant macrophage elevation in the CSOM cochlea at all time points compared to control mice. **N** F4/80-labeled macrophages compared with CD45 positive immune cells showing all similar numbers in all time points except at 3 days. Number of mice per group is in parentheses alongside the timepoint. The data represent mean ± SD. Scale bar = 100 µm

To identify macrophages, we employed the macrophage-specific marker F4/80, which has been used to examine the distribution of mature macrophages in hematopoietic and other tissues of the developing mouse [26]. Macrophages were observed in the stria vascularis, spiral ligament and spiral ganglion neurons in control cochleae (Fig. 5A–C, arrows in 5B). In addition, they were observed beneath the basilar membrane in the scala tympani and the lining of the bone in the scala vestibuli at 3 days (Fig. 5D–F, arrowheads in 5D). The elevation in macrophage numbers in the stria vascularis, spiral ligament and spiral ganglion neurons persisted at 7 days and 14 days after infection (Fig. 5G–L). We calculated the macrophage numbers in the selected area in each cochlear turn (Fig. 5A, dotted line). Macrophage numbers were significantly elevated in the CSOM cochleae compared with control cochleae (Fig. 5M, 3 days vs control, *P* = 0.017; 7 days vs control, *P* < 0.001; 14 days vs control, *P* < 0.001). Significant differences were also observed in CSOM cochleae at both 7 days and 14 days, compared to 3 days (Fig. 5M, 7 days vs 3 days, *P* = 0.019; 14 days vs 3d, *P* < 0.001).

To investigate presence of immune cells other than macrophages in the cochleae, we counted CD45^+^ cells in the same way as F4/80 macrophages. CD45 is a type I transmembrane molecule found on the surface of all nucleated hematopoietic cells and their precursors [27], and labels both innate and adaptive immune cells. CD45^+^ cells were significantly elevated at 3 days, 7 days and 14 days compared to control cochleae (Additional file 3: Fig. S3A–M, 3 days vs control, p < 0.001; 7 days vs control, *P* < 0.001; 14d vs control, *P* < 0.001). The difference between 14 and 3 days CSOM cochleae was also significant (*P* = 0.046). Finally, we compared numbers of CD45^+^ cells and F4/80 macrophages. CD45^+^ cells outnumbered F4/80 macrophages only at 3 days (Fig. 5N, *P* = 0.02). CD45^+^ cells and F4/80 macrophages were present at similar levels at 7 days and 14 days. Collectively, these data demonstrated that macrophages are the major immune cell and the innate immunity is the major immune response in the CSOM cochlea. They are significantly elevated at 7 days and 14 days after inoculation, consistent with the timing of the observed OHC loss.

### Morphological changes of macrophages in the spiral ganglion neuron area

**Fig. 6:**
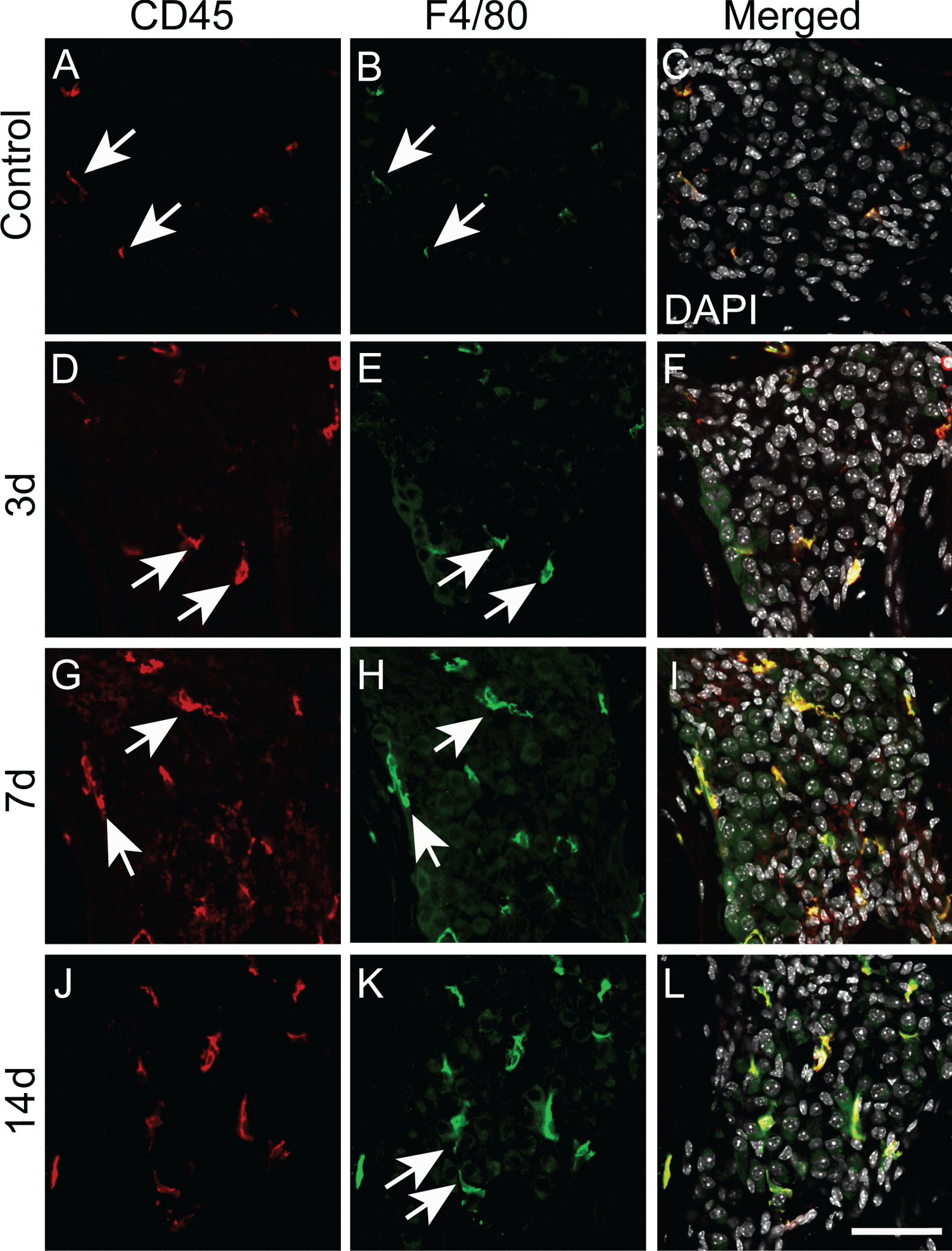
Macrophage morphological changes in the region of spiral ganglion neuron (SGN) in CSOM. Macrophages in cochlear cryosections were labeled with the pan-leukocyte marker CD45 and F4/80. Representative images are shown. Macrophages (arrows) were small with few projections in the control group (**A**–**C**, arrows), and they were bigger and round at 3 days (**D**-**F**, arrows). Macrophages were larger and irregularly shaped at 7 days (**G**–**I**, arrows), and had fine, dendritic projections at 14 days (**J**–**L**, arrows). Scale bar = 50 µm. *n* = 3 per group

Monocytes can differentiate into inflammatory or anti-inflammatory subsets [28, 29]. During tissue damage or infection, monocytes are rapidly recruited to the tissue, where they can differentiate into tissue macrophages or dendritic cells [30, 31]. One likely source for the tissue macrophages in CSOM is from circulating monocytes. Indeed, at 3d post-inoculation, macrophages in the spiral ganglion neuron (SGN) area in CSOM were round and small with fewer dendritic projections compared with the control group (Fig. 6A–F, arrows in 6A, 6B, 6D and 6E), suggesting that they were newly infiltrated monocytes. The morphological changes observed is similar to that in an acoustic injury mouse model [32]. At 7 days post-inoculation, macrophages were irregularly shaped and larger than at 3 days (Fig. 6G–I, arrows in 6G and 6H). Macrophages had fine and short dendritic projections at 14 days post-inoculation (Fig. 6J–L, arrows in 6K). This reveals that macrophages have morphological changes in CSOM, suggesting differentiation from circulating monocytes to tissue-type macrophages in the cochlea.

### Macrophage-associated cytokines are upregulated in CSOM

**Fig. 7:**
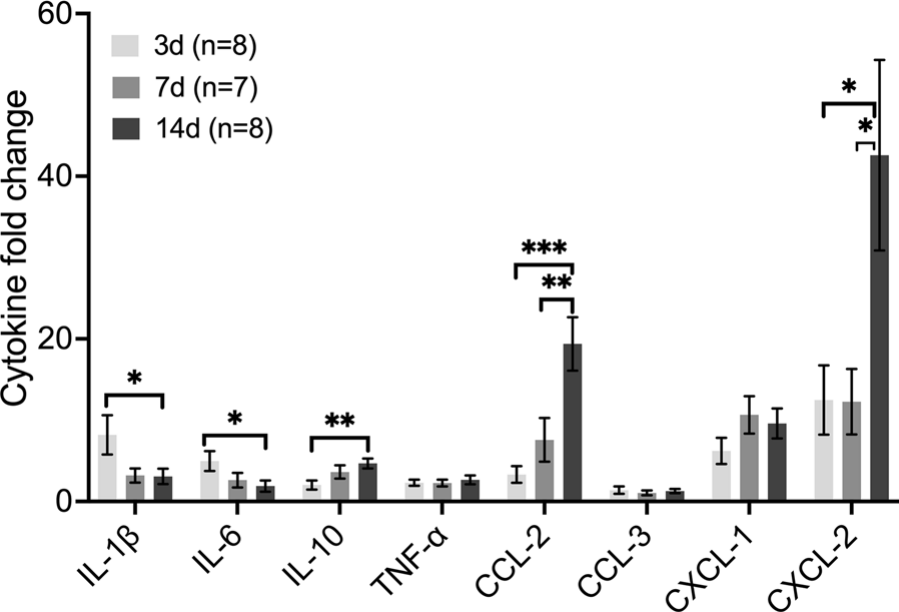
Selected cytokines are upregulated in CSOM. Cochlear selected cytokine expression was assessed via quantitative PCR with normalization to GAPDH and β-actin at 3 days, 7 days and 14 days, and fold-change was quantified relative to control cochlea. The pro-inflammatory cytokines interleukin-1β (IL-1β) and IL-6 displayed increased expression at 3 days compared to 14 days. The anti-inflammatory cytokine IL-10 displayed increased expression at 14 days compared to 3 days. The chemokines CCL-2 and CXCL-2 displayed increased expression at 14 days compared to 7 days and 3 days, respectively. TNF-α, CCL-3 and CXCL-1 remained unchanged in these time points. The mouse number is shown in brackets. The data represent mean ± SEM

To verify cytokine expression, we first performed an immunoassay screen at 7 days after inoculation in the cochlea and middle ear. Cytokine levels in CSOM ears were normalized with levels from contralateral ears. Of 48 analyzed targets, 46 cytokines were upregulated in the CSOM cochlea (Additional file 4: Fig. S4, red). Only IL-33 and CCL-11 were downregulated in the CSOM cochlea, whereas three cytokines including IL33, CCL-11 and IL-2 were downregulated in the CSOM middle ear (Additional file 4: Fig. S4, black). The pattern and level of cytokine expression varied between the CSOM middle ear and cochlea. Among the upregulated cytokines in the CSOM cochlea, we selected 8 candidates (IL-1β, IL-6, IL-10, TNF-α, CCL-2, CCL-3, CXCL-1 and CXCL-2) that either were increased in previously described acute otitis media models [33, 34], models of noise-induced hearing loss [35, 36] or associated directly with macrophage function. We performed RT-qPCR to assess levels of these 8 cytokines. All selected cytokines were upregulated at 3 days, 7 days, and 14 days compared with control cochleae (Fig. 7). The pro-inflammatory cytokines IL-1β and IL-6 were highly expressed at 3 days and decreased at 14 days (both *P* ≤ 0.05). The anti-inflammatory cytokine IL-10 was upregulated at 14 days compared with 3 days (*P* < 0.005). CCL-2 was elevated significantly at 14 days compared to both 3 days (*P* < 0.001) and 7 days (*P* < 0.005). Significant upregulation at 14 days compared to 3 days and 7 days was also observed for CXCL-2 (14 days vs 3 days, *P* < 0.05; 14 days vs 7 days, *P* < 0.05). TNF-α, CCL-3 and CXCL-1 displayed no changes in expression level over time. These data indicate that inflammatory cytokines are elevated in CSOM at both the protein and mRNA levels.

## Discussion

SNHL in CSOM has been well described as a clinical problem [10, 11, 37–40]. Now with a validated animal model mimicking the human disease, we have an opportunity to begin to understand in vivo mechanisms of SNHL in CSOM [13]. We observed, as in human CSOM, a pattern of OHC loss occurring in the high-frequency region of the cochlea.

One possible mechanism for CSOM-mediated OHC loss is direct bacterial injury. We found that while PA is observed in the middle ear, it is not in the cochlea at any experimental time point. Therefore, PA is unlikely to directly invade the inner ear to cause OHC loss in this model, highlighting a distinct difference between inner ear hearing loss caused by acute and chronic infections of the middle ear.

Another possible mechanism for CSOM-mediated OHC loss in this model is bacterial production of ototoxic substances, including LPS. To investigate whether live PA were required for cochlear injury, we inoculated inactivated PA using dry heat, a method known to preserve LPS [24]. Heat-inactivated PA inoculation did not cause HC loss. Further to this, inoculated stationary phase PA supernatant that likely contains the higher concentrations of potential PA ototoxins did not result in HC loss. While we do not rule out the potential for any ototoxin to enter the inner ear, this study shows any concentrations in the inner ear are likely to be below threshold for inducing injury. These results suggest that ototoxins do not mediate HC loss directly in CSOM.

Further supporting this conclusion, we did not observe changes in permeability of structural compartments. The round window membrane (RWM) is the main barrier between the middle ear and inner ear and is semipermeable, known to allow passage of a wide range of substances including ototoxins [41]. It is controversial whether RWM permeability increases or decreases in middle ear infection with contradictory findings in similarly designed studies [12, 42]. However, we did not observe generalized breakdown of the RWM.

Finally, immune cell-mediated damage might explain our findings in this model of CSOM. We and others previously showed that CSOM contains a predominance of neutrophils in the middle ear [3, 25, 43]. This current study showed a paucity of neutrophils in the inner ear and the timing observing these neutrophils was not in keeping with the observed OHC loss, ruling this cell out as the main driver for SNHL in CSOM.

Instead, we observed macrophages correlated in a pattern matching SNHL in CSOM. Macrophages have been studied broadly in other forms of hearing loss, such as ototoxic drug exposure, NIHL and age-related hearing loss (ARHL) [44–46]. There is a known resident CD45+ macrophage population with a morphology featuring multiple long projections in the apex and an amoeboid shape in the base [44].

The exact role of macrophages in the cochlea remains unclear. Depending on the activation status, they seem to have different roles. Cochlear macrophages are thought to be the major executor for cochlear immune homeostasis [32, 45, 47, 48]. Conversely, perivascular resident macrophages in the stria vascularis are essential for the integrity of intrastrial fluid–blood barrier and cochlear function [49, 50]. Macrophages have been found to engulf hair cell debris in lesioned utricles [51] and to mediate a wound healing response in the context of injury [52, 53]. However, macrophages can also cause cell death by the release of proinflammatory factors and cytotoxic products including various interleukins, tumor necrosis a, quinolinate, reactive oxygen inter mediates, and nitrous oxide [54, 55]. The migrating macrophages may therefore be a more likely source of secondary damage to the cochlea. Thus, it is possible that the proinflammatory factors produced by these macrophages promote the hair cell damage. To this point, the pattern of injury we observed mirrors other forms of inner ear injury, including NIHL and ototoxic drug hearing loss [56] where radical oxygen species (ROS) production mediate damage to basal region OHCs [57–63].

Macrophage production of cytokines promoting further inflammation linked to ROS-mediated cell injury [64, 65]. Indeed, we observed increased levels of several cytokines known to mediate inner ear injury, including IL-6 and IL-1β [29, 35, 66, 67]. Conversely, inhibiting IL-6 expression suppressed inflammation in the inner ear [68]. In contrast to pro-inflammatory cytokines, we observed later increases in anti-inflammatory cytokines and chemokines, including IL-10, CCL2 and CXCL-2. Our finding of distinct patterns of cytokine expression in cochlea versus the middle ear suggests that the inner ear represents its own separate immune area with an active immune response to the middle ear infection. Whether macrophages, their cytokines, or ROS are responsible for inciting the OHC injury seen in this model or a merely involved in the repair process after damage requires further investigation.

## Conclusions

Our study of CSOM cochlea revealed significant OHCs loss in the cochlear at 14 days following infection. This pathogenesis required live bacterium in the middle ear and was associated with increased numbers of macrophages. Further studies are required to determine if the elevated macrophages derived from circulation or resident macrophages and to relate these findings to human disease.

## Supplementary Information

**Additional file 1: Figure S1.** Method of PA-inoculation to create CSOM. After creating a subtotal tympanic membrane perforations, the PC inoculum was directly injected into the middle ear (A). We then grade the CSOM infection as previously published (13). At 3d, no visible effusion was present (B, grade II), while grade III and grade IV CSOM involving suppuration and mucosal disease were observed at 7d and 14d, respectively (C-D). This Figure was created with BioRender.com.

**Additional file 2: Figure S2.** Live PA are required for OHC loss in CSOM. Representative whole-mount sections of the cochlear base stained with myosin-VIIa demonstrated no OHC loss at 21 days (21d) following inoculation of heat-inactivated PA (A) or toxin-containing supernatant from stationary phase PA (B). There were 3 mice in each group. Scale bar=100 µm.

**Additional file 3: Figure S3.** CD45 positive immune cells are significantly increases in the CSOM cochlea. Cochlear cryosections were stained with pan-leukocyte marker CD45 (red) and were counted by the same method as in Fig.5 from control mice (A-C) to CSOM mice at various time point 3d (D-F), 7d (G-I) and 14d (J-L). Myosin VIIa staining (white) labels HCs in the cochlea. Significant CD45 positive cells elevation in the CSOM cochlea at all time points compared to control mice, and there was also a significant elevation at 14d compared with 3d (M). Number of mice per group is in parentheses alongside the timepoint. The data represent mean±SD. Scale bar=100µm.

**Additional file 4: Figure S4.** A broad range of cytokines are upregulated at 7d on immunoassay analysis. The average ratio of expression in the left (L), infected CSOM ear compared to the contralateral non-infected ear (R). 46 of 48 traget cytokines were upregulated in the cochleae (red) and 45 of 48 were upregulated in the middle ears (black). The data (mouse number =3) represent mean ± SD.

**Additional file 5: Figure A5**. Schematic diagram of experimental design. The animal numbers are present in the brackets at each time point. PC: Persister cells. PA: Pseudomonas aeruginosa. PBS: Phosphate-buffered saline. IVIS: in vivo imaging system. RT-PCR: real time PCR. WMI: Whole mount immunohistochemistry. CSI: Cyrosection immunohistochemistry. C assay: Cytokine assay. C qPCR: Cytokine qPCR.

## Data Availability

All data generated or analyzed during this study are included in this published article [and its supplementary information files].

## Abbreviations

CSOM: Chronic suppurative otitis media
PA: *Pseudomonas aeruginosa*
PCs: PA persister cells
SNHL: Sensorineural hearing loss
OHC: Outer hair cell
IHC: Inner hair cell
RWM: Round window membrane
NIHL: Noise-induced hearing loss
ROS: Radical oxygen species
