# Supplementary figures and images for: Chronic suppurative otitis media causes macrophage-associated sensorineural hearing loss
Source: J Neuroinflammation. 2022 Sep 12;19:224. doi: 10.1186/s12974-022-02585-w (PMC9465898; doi:10.1186/s12974-022-02585-w)

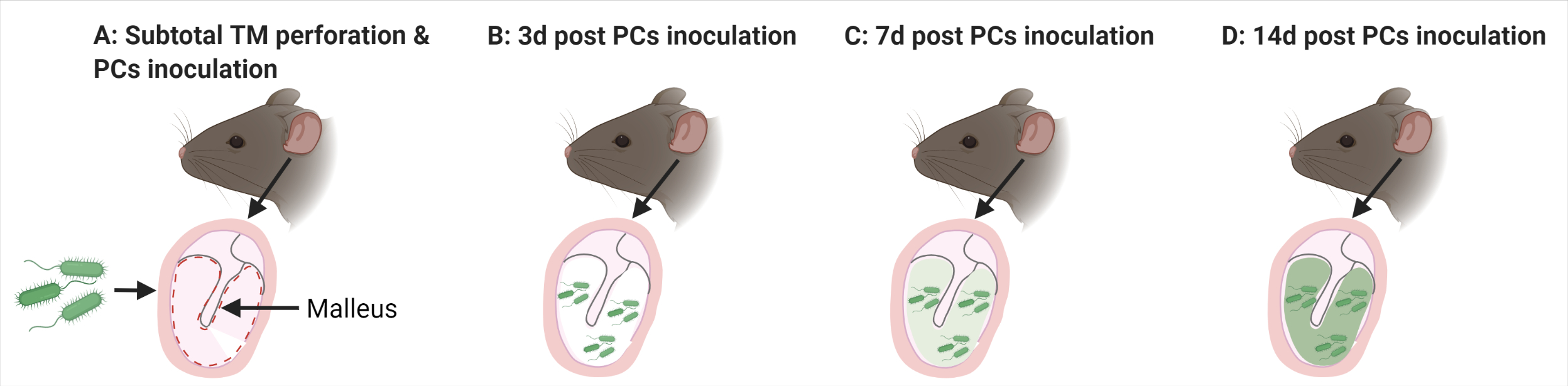

Supplement: Supplementary file 1 — Additional file 1: Figure S1. Method of PA-inoculation to create CSOM. After creating a subtotal tympanic membrane perforations, the PC inoculum was directly injected into the middle ear (A). We then grade the CSOM infection as previously published (13). At 3d, no visible effusion was present (B, grade II), while grade III and grade IV CSOM involving suppuration and mucosal disease were observed at 7d and 14d, respectively (C-D). This Figure was created with BioRender.com. [file 12974_2022_2585_MOESM1_ESM.jpg]

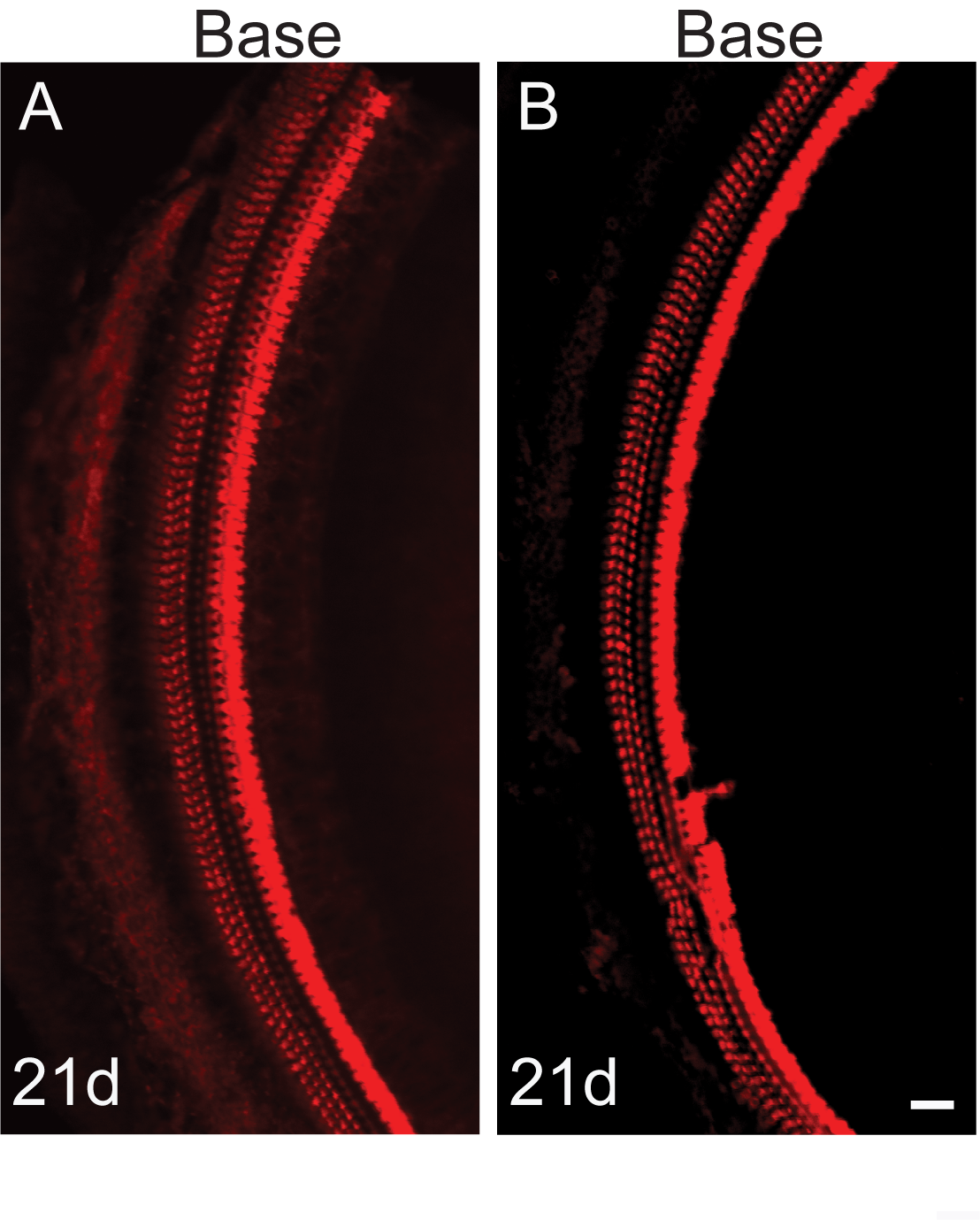

Supplement: Supplementary file 2 — Additional file 2: Figure S2. Live PA are required for OHC loss in CSOM. Representative whole-mount sections of the cochlear base stained with myosin-VIIa demonstrated no OHC loss at 21 days (21d) following inoculation of heat-inactivated PA (A) or toxin-containing supernatant from stationary phase PA (B). There were 3 mice in each group. Scale bar=100 µm. [file 12974_2022_2585_MOESM2_ESM.tif]

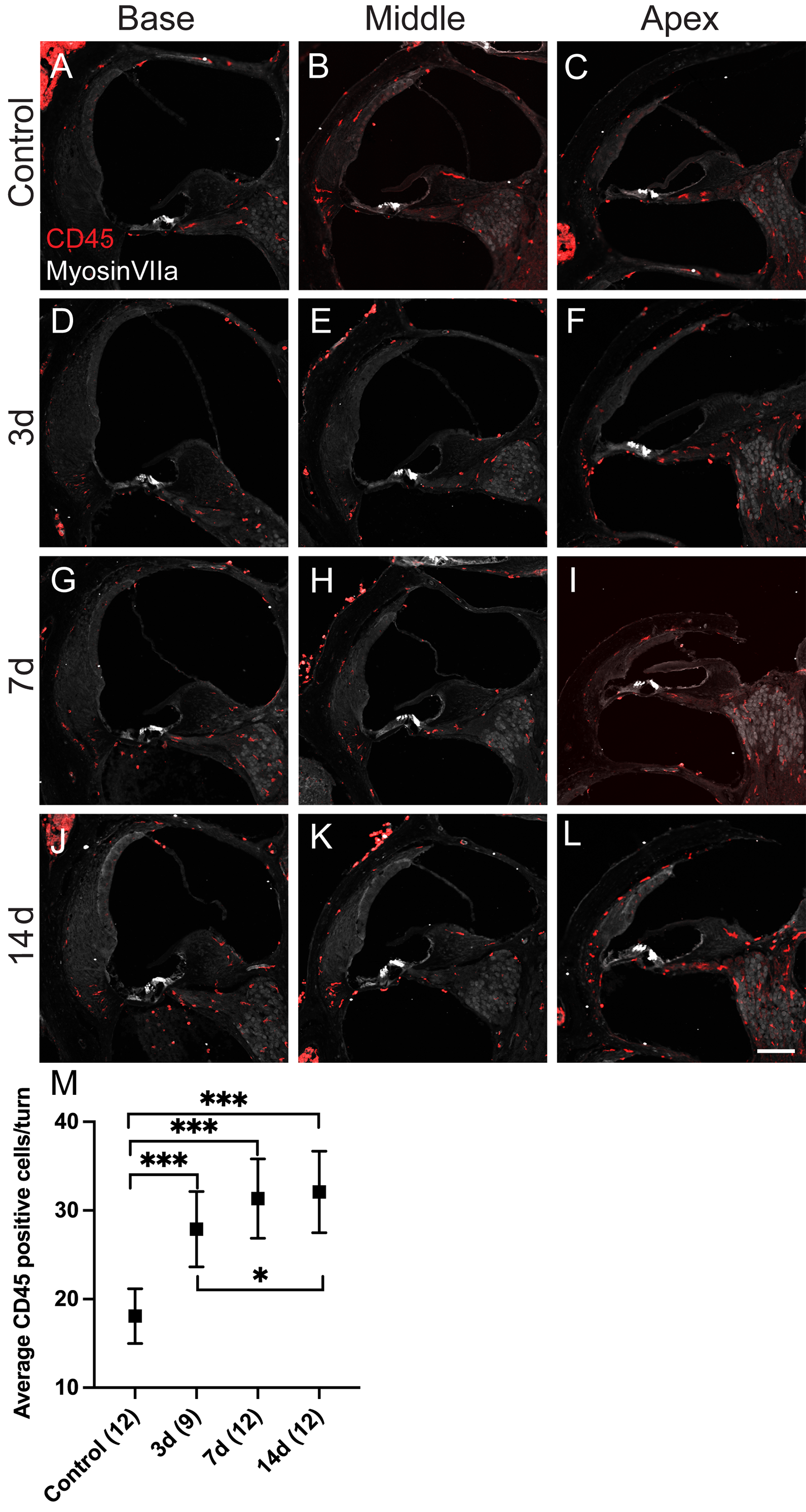

Supplement: Supplementary file 3 — Additional file 3: Figure S3. CD45 positive immune cells are significantly increases in the CSOM cochlea. Cochlear cryosections were stained with pan-leukocyte marker CD45 (red) and were counted by the same method as in Fig.5 from control mice (A-C) to CSOM mice at various time point 3d (D-F), 7d (G-I) and 14d (J-L). Myosin VIIa staining (white) labels HCs in the cochlea. Significant CD45 positive cells elevation in the CSOM cochlea at all time points compared to control mice, and there was also a significant elevation at 14d compared with 3d (M). Number of mice per group is in parentheses alongside the timepoint. The data represent mean±SD. Scale bar=100µm. [file 12974_2022_2585_MOESM3_ESM.tif]

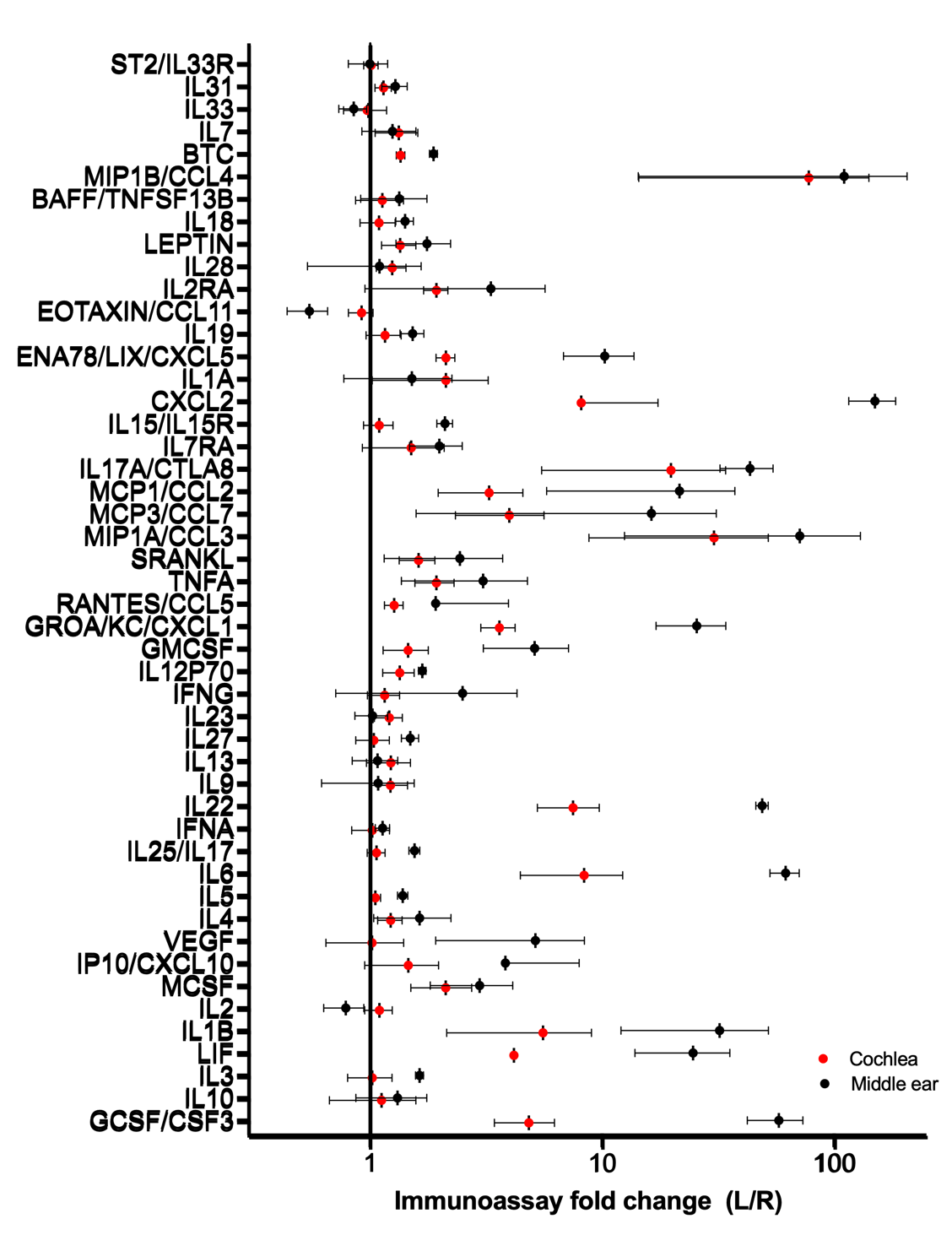

Supplement: Supplementary file 4 — Additional file 4: Figure S4. A broad range of cytokines are upregulated at 7d on immunoassay analysis. The average ratio of expression in the left (L), infected CSOM ear compared to the contralateral non-infected ear (R). 46 of 48 traget cytokines were upregulated in the cochleae (red) and 45 of 48 were upregulated in the middle ears (black). The data (mouse number =3) represent mean ± SD. [file 12974_2022_2585_MOESM4_ESM.tiff]

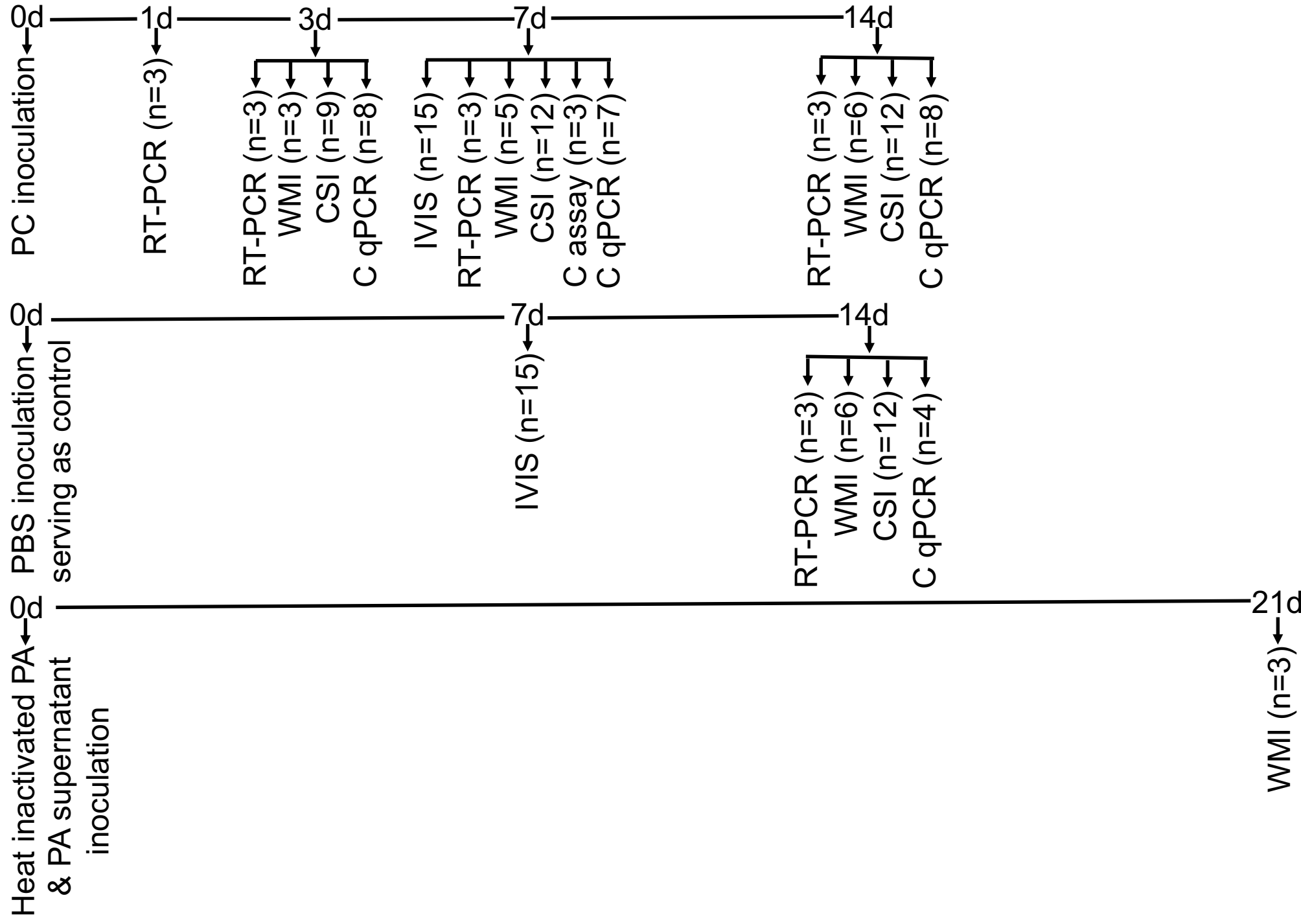

Supplement: Supplementary file 5 — Additional file 5: Figure A5. Schematic diagram of experimental design. The animal numbers are present in the brackets at each time point. PC: Persister cells. PA: Pseudomonas aeruginosa. PBS: Phosphate-buffered saline. IVIS: in vivo imaging system. RT-PCR: real time PCR. WMI: Whole mount immunohistochemistry. CSI: Cyrosection immunohistochemistry. C assay: Cytokine assay. C qPCR: Cytokine qPCR. [file 12974_2022_2585_MOESM5_ESM.pdf]
